# Prevalence of hypertensive retinopathy and its associated factors among adult hypertensive patients attending at Comprehensive Specialized Hospitals in Northwest Ethiopia, 2024, a multicenter cross-sectional study

**DOI:** 10.1186/s40942-025-00631-2

**Published:** 2025-02-17

**Authors:** Yitayal Abebe Gudayneh, Abebech Fikade Shumye, Abebech Tewabe Gelaye, Melkamu Temeselew Tegegn

**Affiliations:** 1 Department of Optometry, School of Medicine, College of Medicine and Health Science, University of Gondar, Gondar, Ethiopia; 2 Department of Clinical Pharmacy, School of Pharmacy, College of Medicine and Health Science, University of Gondar, Gondar, Ethiopia

**Keywords:** Prevalence, Hypertensive retinopathy, Adult, Hypertensive patients, Northwest Ethiopia

## Abstract

**Introduction:**

Hypertensive retinopathy refers to changes in the retinal microvasculature resulting from elevated blood pressure, and the global burden ranges from 2 to 85%. However, there was no evidence on prevalence and associated factors of hypertensive retinopathy among adult hypertensive patients in the study area even in Ethiopia.

**Objective:**

The aim of this study was to investigate prevalence of hypertensive retinopathy and associated factors in adult hypertensive patients attending at Comprehensive Specialized Hospitals in Northwest Ethiopia in 2024.

**Methods:**

Multicenter hospital-based cross-sectional study was conducted in Northwest Ethiopia Comprehensive specialized hospitals from June, 07 to August, 07, 2024. A multistage sampling technique with an interval of 3 was applied to select 696 study participants. Data were collected through personal interviews, review of medical records and eye examinations. Data were entered into the Kobo Toolbox and then transferred to STATA version 17 for analysis. Bivariable and then multivariable binary logistic regression models were fitted to determine factors associated with hypertensive retinopathy. Variables with a P-value of less than 0.05 at multivariable analysis were considered statistically significant.

**Result:**

A total of 696 (95.34%) participants were included in the study. The prevalence of hypertensive retinopathy was 57.47%(95%CI: 53.75, 61.10). Age > 74 years (AOR = 4.24, 95%CI = 1.54,11.64), heart disease(AOR = 5.38, 95%CI = 1.86,15.58), duration of hypertension > 5years (AOR = 12.66, 95%CI = 3.88,41.29), dyslipidemia (AOR = 3.44,95%CI = 1.59–7.45), uncontrolled current levels of hypertension (AOR = 40.03, 95%CI = 17.19,93.18), poor adherence of hypertensive medications (AOR = 1.84, 95%CI = 1.12,3.03) and diabetes (AOR = 3.56, 95%CI = 1.49,5.99) were positively associated with hypertensive retinopathy.

**Conclusion:**

-The prevalence of hypertensive retinopathy is high among systemic hypertensive patients seen in Northwest Ethiopia comprehensive specialized hospitals and independently associated with older age, longer duration of hypertension, heart disease, diabetes, dyslipidemia, poor adherence of hypertension medications and uncontrolled hypertension. Early diagnosis and treatment of hypertension was recommended to prevent target organ complications.

## Introduction

According to World health organization; systemic arterial hypertension (HTN) is defined as a systolic pressure greater than 140 mmHg and/or a diastolic pressure greater than 90 mmHg. It also estimates that; 1.13 billion people worldwide have HTN and fewer than 1 in 5 people with HTN have under control their blood pressure which declared as the cause for more than 10 million deaths per year [1]. This systemic devastating vascular disease had been progressively increasing in the study area [2, 3] as well as in Ethiopia [4, 5]. It is also a well-known risk factor for other diseases; called hypertension-mediated target organ damage (TOD), such as stroke, disability, myocardial infarction, hypertensive retinopathy(HR) and others [6].

HTN affects the eye causing 3 types of ocular damage: choroidopathy, retinopathy, and optic neuropathy [7]. HR refers to changes in retinal microvasculature that occurs due to elevated BP [8, 9]. It is a visible manifestation of hypertensive vascular damage in which millions of people in the world are suffering with systemic morbidity and mortality due to TOD [8–10].

The common clinical signs of HR are focal arteriolar narrowing, arterio-venous nicking (AVN), micro-aneurysms, soft exudates, blot hemorrhages, flamed-shaped hemorrhages, cotton wool patches and optic disc edema [11, 12]. In addition, HR may also associate with various ocular morbidities; such as sub-conjunctival hemorrhage, retinal vein occlusion; retinal artery occlusion, ischemic optic neuropathy and cranial nerve palsy [13–16].

Duration of HTN, poor control of BP, older age, smoking, concurrent hyperlipidemia, high plasma level of endothelin-1 and family history of HTN was significantly associated with HR [14–19]. Furthermore, serum uric acid concentration levels were significantly associated with HR [20].

Studies showed that the magnitude of HR ranges from 2 to 85% [9, 21–23]. Pakistan 85% [21] and 56% [24], Bangladesh 29.9% [25], China 77.1% [26] and 76.6% [27], Nepal 83.7% [28], 56.5% [29] and 12.6% [30] Iran 39.9% [11], India 12% [31], 28.5% [32], 62.25% [33], 49.33% [34], and 21% [35] USA 6.7% [36],Texas 51.6% [37], Denmark 8.3% [38] and 20.3% [39], Cameroon 16% [40], Kenya 23.3% [41], Democratic Republic of Congo 83.6% [42], Tanzania 70.1% [22], Malawi 75% [43], Nigeria 71% [44] were some studies which reflect burdens of HR.

In most patients; HR does not cause vision loss, if HTN is treated. However, if the BP remains untreated, it can lead to vision loss within a short period. The cause of vision loss might be either due to retinal pigmentary changes or secondary to optic atrophy, neither of which is reversible [13–16, 45]. Visual impairment due to HR can also be gradual and painless [46] but has a devastating effect on the working age group in the world affecting the daily lives of millions of people [19]. The severity of HR ranges from grade I(arterial narrowing) to grade IV(disc edema) [47].

Due to the high prevalence of HR and its associated increased morbidity and mortality, the economic cost of hypertensive-retinopathy diseases was estimated at $76.6 billion per year in 2010 [48]. It also affects one’s quality of life, independence, and mobility [49]. In Northwest Ethiopia the magnitude of visual impairment among hypertensive patients was reported as 32.4% [49].

Raising prevalence of HTN and clinically observed HR with no evidence of prevalence and factors associated with HR in the study area, even in Ethiopia were the primary reasons for conducting this study. The information about the magnitude and associated factors could impact subsequent disease treatment and management plans [9]. Therefore, this study aimed to assess the prevalence of HR and its associated factors among adult hypertensive patients attending Comprehensive Specialized Hospitals in Northwest Ethiopia, 2024. Furthermore, this study will give baseline evidence for further HR study in Ethiopia.

## Methods and materials

### Study design

A multi-centered hospital-based cross-sectional study.

### Study area and period

This study was conducted at University of Gondar, Felege Hiwot, and Tibebe Ghion Comprehensive Specialized Hospitals in Northwest Ethiopia from June 07 to August 07, 2024.

All comprehensive specialized hospitals had ophthalmic departments staffed by ophthalmologists, optometrists, and ophthalmic nurses. All hospitals uniformly do a dilated fundus examination with a + 90D Volk lens to examine the fundus.

### Source and study population

#### Source population

All adult hypertensive patients who were on hypertensive medical care at Northwest Ethiopia Comprehensive Specialized Hospitals.

#### Study population

All hypertensive patients aged ≥ 18 years old who were on hypertensive medication with HTN duration ≥ 3months or faced with hypertensive crisis at University of Gondar, Tibebe Gion, and Felege Hiwot Comprehensive Specialized Hospitals and available during the data collection period.

### Inclusion and exclusion criteria

#### Inclusion criteria

Adult hypertensive patients on hypertensive medical care, HTN duration ≥ 3months or faced with complication available during the data collection period were included.

#### Exclusion criteria

Adult hypertensive patients who were unable to answer the questionnaire because of speech or mental health problem, patients who were admitted to the inpatient unit due to a serious illness that prevented slit-lamp examination, patients with dense central media opacities which affects visualization of the funds and patients with a shallow anterior chamber angle, HTN duration < 3months were excluded from the study.

### Sample size determination

#### Sample size determination for objective one

The sample size was calculated by using single population proportion formula within the following assumptions:


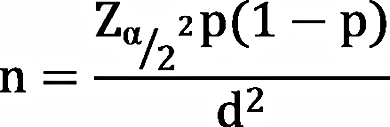


Where,

n = the required sample size,

Z = Value of z statistic at 95% confidence level = 1.96,

P = expected proportion to HR among hypertensive patients were assumed to be 0.5 (50%). This was because, as far as exhaustive search, there was no study previously done on HR prevalence in Ethiopia. However it was not taken from other settings since socio-demographic, health service delivery; diagnostic material difference and clinical variation were considered.

d = margin of error, which was 5% (0.05), therefore, the calculated sample size was 385.

#### Sample size determination for objective two

Family history of hypertension [19], uncontrolled blood pressure and treatment modality of HTN [50],were factors positively associated with HR and were used to calculate the sample size of objective two through Epidemiological Information (EPI INFO) 7 computer software (Table 1).

**Table 1:** Sample size determination for objective two by considering the family history of hypertension, uncontrolled blood pressure and treatment of HTN as the factor of hypertensive retinopathy

Variable		Hypertensive retinopathy					Power and confidence level	Sample size
		Yes	No	Crude odds ratio	Ratio of unexposed to exposed	Percentage of outcome in the unexposed group		
Family history of hypertension	Yes	79	67	1.75	0.74	54.1%	80% power 95% confidence level	442
	No	33	49					
Uncontrolled blood pressure	Yes	17	51	0.117	2.96	74%		
	No	20	7					50
Treatment of hypertension	Yes	23	49	0.300	1.9	60.9%		116
	No	14	9					

Finally, Sample size determined by using the family history of hypertension was selected since it was larger than the first objective and sample size determined based on uncontrolled BP and treatment of HTN. *n*=442×10&×1.5, *n*=730.

By considering 10% non-response rate and a design effect of 1.5, the final planned sample size was 730.

### Sampling technique and procedures

A multistage sampling technique was employed during sampling process. Among 5 comprehensive specialized hospitals in Northwest Ethiopia, 3 hospitals were selected by using lottery method. Based on the number of patients attending at chronic clinic, proportional allocation of the desired sample was made among the three hospitals. The logbooks of three hospitals on average showed that, about 2264 hypertensive patients were attended in previous 2 years with similar data collection period (June 07 to August 07/2022 and 2023). By taking this statistics; proportional allocation were done among those hospitals.

**Fig. 1:**
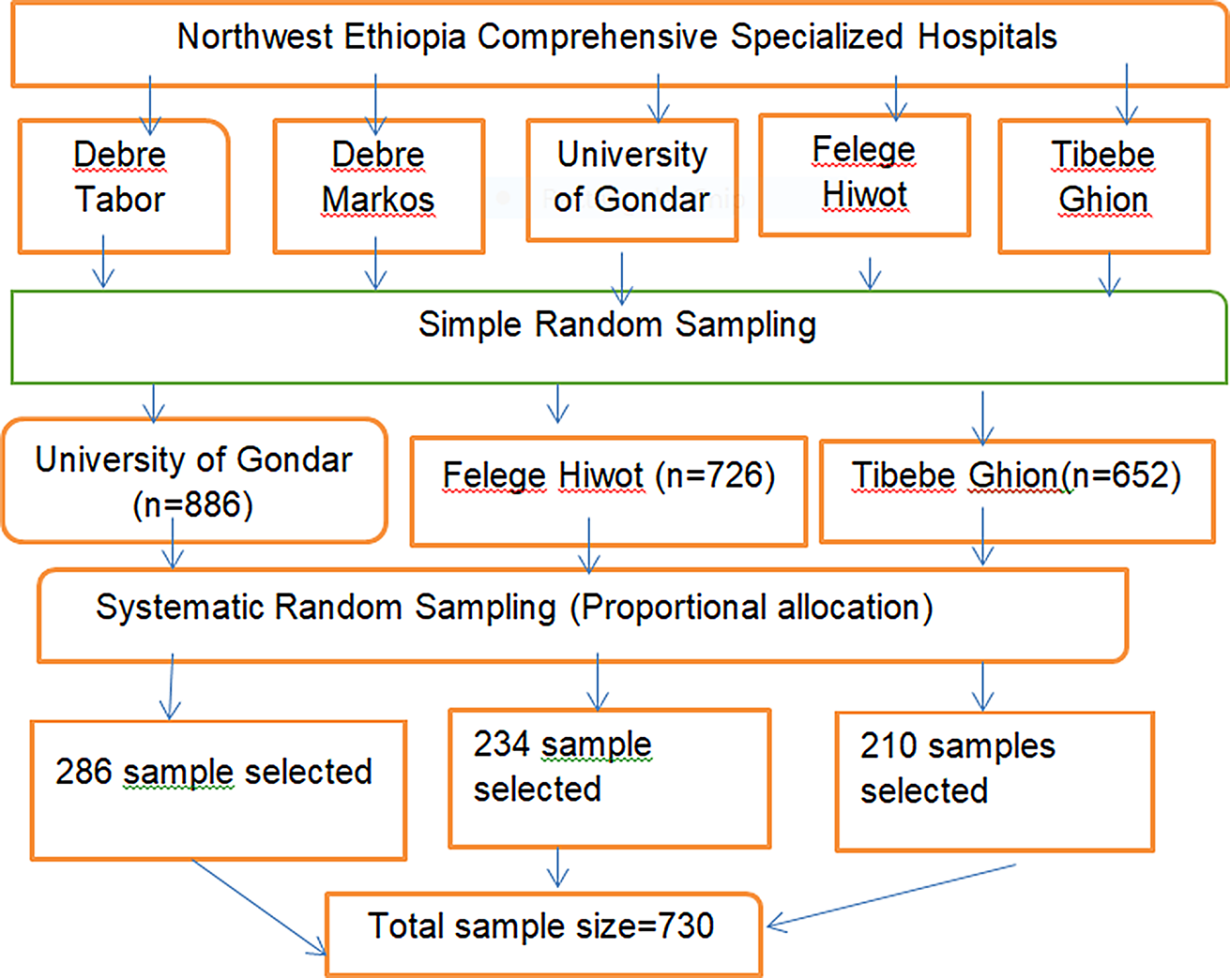
A diagram shows the sampling procedure and sampling techniques of study participants attending at Comprehensive Specialized Hospitals in Northwest Ethiopia, 2024

Finally, systematic random sampling technique with an interval of 3 was applied to select the study participants. The sampling interval were calculated by dividing the expected number of hypertensive patients who had come to the hypertensive clinic last 2years in the same months with the data collection period by the required sample size. The k^th^ interval calculation was thus, K = N/*n* = 2264/730 = 3.1. Again simple random sampling (Lottery method) was applied to draw the 1st sample of the first 3 participants and continue with every third participant (Fig. 1).

### Study variables

#### Dependent variable

Hypertensive retinopathy.

### Independent variables

#### Socio-demographic variables

age, sex, residence, marital status, educational status, occupational status, family average monthly income, health insurance, family history of hypertension.

#### Behavioral factors

cigarette smoking, physical exercise, Checking blood pressure regularly, hypertensive medication adherence.

#### Systemic comorbidities factors

Atherosclerosis, diabetes mellitus, chronic heart disease, chronic kidney disease, dementia, cognitive impairment, dyslipidemia.

#### Clinical factors

-Duration of hypertension, severity of disease (uncontrolled or untreated), body mass index, duration hypertensive medication, serum uric acid concentration, high plasma level of endothelin-1,

#### Ocular related factors

History of cataract surgery, History of eye examination, awareness of hypertension affects the eye/s, physicians’ advice to do eye checkup, and having controlling BP to preserve vision.

### Operational definitions

**Hypertensive Retinopathy (HR)**: was diagnosed by using a + 90D volk lens under mydriasis using tropicamide 1% eye drop. It was categorized as HR positive when at least one of the following clinical findings was observed at least in one eye; detectable arterial narrowing, AVN, retinal hemorrhage and/or exudates and disc swelling (papilledema), silver wiring, vascular tortuosity, and negative when there was none of the above signs were present. Furthermore; HR was recorded Stage 1: Widening of the arteriole light reflex;

Stage 2: Stage 1 + arteriovenous crossing sign.

Stage 3: Copper wiring of arterioles (copper-coloured arteriole light reflex).

Stage 4: Silver wiring of arterioles (silver-coloured arteriole light reflex) modified Scheie Classification [47, 51].

**Body mass index (Kg/m**^**2**^**)-**was calculated as weight (kg) divided by height in square meters (m2) and was graded according to the World health organization classification. When A BMI of < 18.5 was underweight, a BMI of 18.5–24.9 kg/m2 was normal, BMI in between 25.0 and 29.9 kg/m2 it was recorded as overweight, while BMI more than 30 kg/m2 was obese- defined [52].

#### History of eye examination

Participants received an eye examination at least once within a year after being diagnosed as hypertensive before the data collection period [53].

#### Physical exercise

This can be defined poor; when regular exercise was less than 150 min (3–5 days) of work per week and it was considered to have good regular exercise when greater than 150 min(≥ 5days)of work per week [54].

#### Duration of HTN

Was categorized based on interquartile range as ≤ 3 years, 3–5 years, 5–8 year and > 8 years but the last category had less 5 cells in cross tabulation and it was merged in third categories.

#### Smoking cigarette

A person was grouped under smoker when at least he/she smokes one stick per day [55].

#### Medication adherence

An activity a patient used their hypertensive medications as indicated by their physicians. Adherence to hypertensive medication was assessed with a six-item compliance scale and scored 1 point for each positive response, so that a higher score indicated a lower level of compliance. A score of 3 or more positives was taken as poor adherence [56].

#### Current levels of hypertension

It was grouped under uncontrolled when (systolic BP ≥ 140mmHg or diastolic BP ≥ 90mmHg) [57].

#### Dyslipidemia

It was defined when total cholesterol (> 200 mg/dl), triglycerides (> 150 mg/dl), low density lipoprotein (> 160 mg/dl) [58].

#### Age

It was categorized as 19–51 years, 52–63 years, 64–74 years, and age > 74 years based on interquartile range.

#### Visual acuity

Presenting distance visual acuity status of the study participants was categorized as normal visual acuity (if a presenting visual acuity(PVA) was better than 6/12), mild visual impairment(if a PVA was < 6/12 to ≥ 6/18), moderate visual impairment(if a PVA was < 6/18 to ≥ 6/60), severe visual impairment(if a PVA was < 6/60 to ≥ 3/60), and blind (if a PVA was less than 3/60) based on International Calcification of Disease 11th definition of visual impairment [59].

### Data collection technique, instruments and personnel

Data were collected through face-to-face interviews for socio demographic informations, medical record reviews for recording medications and laboratory profile to check whether it was done before ¾ monthes or not, and an ocular examination. A total of 9 data collectors were involved during the process. A face-to-face interview, review of a medical record, and measurement of height and weight of the study participants were done by trained geneneral nurses while an ocular examination was performed by senior clinical optometrists and ophthalmologists.

A face-to-face interview was conducted using a pre-tested and structured questionnaire, which consists of information on socio-demographic characteristics, behavioral data, past ocular history and adherance to hyperensive medication.

A structured questionnaire was adapted from the previous similar literatures [19, 60]. General nurses was recording medical, socio-demographic factors and behavioral characteristic variables, measure blood pressure and relevant tests as requested. optometrists for visual acuity testing and recording, anterior and posterior segment examination while ophthalmologists involved for diagnosis of HR. Clinical data such as current level of hypertention, duration of hypertention, treatment modality, and systemic co-morbidities like diabetes, heart disease, dyslipidemia, atherosclerosis, and kidney disease were recorded from each study participants’ medical record. Weight was measured using a balance-beam scale, and height was measured using a wall-mounted stadiometer with participants in their underwear and without shoes. After completing the interview, all the study participants underwent a comprehensive ocular examination. A presenting distance visual acuity of the study participants was measured in each eye using a snellen acuity chart at distance of 6/3 meters in optimal room illumination.

Anterior segment eye examination followed by poster segment eye examination in dilated pupil using 1% tropicamide eye drop was performed for each eye using slit-lamp biomicroscope with a 90-dioptre volk lens to diagnois of hyperetensive retinopathy. Vital signs(blood pressure) were measured from all study participants.

### Data quality assurance

To ensure the qualty of of this study, the data collection tools were pretested on 5% of the sample size at Debre Tabor comprehensive specialized hospital. English version of data collection tools was translated to Amharic for the purpose of simplicity and to communicate easily with study participants and after the required data were collected. Then, training was given for the data collectors on how to collect the data by principal investigator and supervision was made. Then after it was returned back to keep its original meaning. Furthermore, the collected data were checked for completeness at the end of the day to ensure data quality.

### Data processing and analysis

All the necessary information collected from the study participants were entered into kobo toolbox and then transferred to STATA version 17 for analysis. Multi-collinearity was checked using the variance inflation factor and tolerance and no significant relationship was detected between the predictor variables (VIF = 2.25). Proportions and summary statistics such as median and standard deviation were calculated for the descriptive data.

Bivariable followed by a multivariable binary logistic regression were fitted to determine factors associated with HR. Variables with a P-value less than 0.2 in the bivariable analysis were entered into a multivariable logistic regression. The strength of the association between dependent and independent variables was expressed by an adjusted odds ratio (AOR) with a 95% confidence interval (CI). The model fitness was checked by estat gof and the p- value was 0.314(> 0.05). Variables having p-values of less than 0.05 in multivariable logistic regression analysis were declared as associated factors of HR.

## Results

### Socio-demographic and behavioral characteristics of the study participants

Six hundred ninety six study participants were involved in this study, with a response rate of 95.34%. The median age of the study participants was 63years with IQR [50, 73]. Among the study participants, 374(53.74%), were male, 450(64.66%) were urban resident, 395(56.8%) had formal educational status, 5(0.72%) were smokers, 261(37.5%) had poor physical exercise habit, 166(23.85%) had no health insurance, 136(19.54%) had family history of HTN, 591(84.91%) had check their BP regularly (Table 2).

**Table 2:** Socio-demographic and behavioral characteristics of adult hypertensive patients attending at Comprehensive Specialized Hospitals in Northwest Ethiopia, 2024(*n* = 696)

Variables	Frequency	Percent
**Occupational status**		
Farmer	225	32.3
Retire	104	14.9
Non-government employee	97	13.9
House wife	89	12.9
Government employee	79	11.4
Merchant	44	6.3
**Others**	58	8.3
**Marital status**		
Unmarried	29	4.20
Married	534	76.7
Divorced	32	4.60
Widowed	101	14.50
**Family average monthly income (Ethiopian birr)**		
500–4148	174	25
4149–8000	185	26.6
8001–12,000	192	27.6
>12,000	145	20.8

**Note: Others** includes driver, Broker, monk, oustaz, hotelier, mechanic, family dependent)

### Clinical and systemic characteristics of the study participants

Among the study participants; 688(98.85%) were on antihypertensive medication, 474 (68.1%) had controlled their HTN, 299(42.96%) had hypertensive related complications. hypertensive related complications were; 233(33.48%) had DM, 180(25.86%) had dyslipidemia, 139(19.97%) had heart disease, 112 (16.09%) had stroke, 81(11.64%) had kidney disease and 26%(3.34%) had hypertensive related complications. There were two dementia and cognitive impaired hypertensive patients with psychotic medication and they were positive for HR (Table 3).

**Table 3:** Clinical and systemic characteristics of adult hypertensive patients attending at Comprehensive Specialized Hospitals in Northwest Ethiopia, 2024(*n* = 696)

Variables	Frequency	Percent
**Adherence to Hypertensive medication**		
Good	374	53.74
Poor	322	46.26
**History of atherosclerosis**		
No	670	96.26
Yes	26	3.74
**History of kidney disease**		
Yes	81	11.64
No	615	88.36
**Body mass index(kg/m** ^**2**^ **)**		
<25	483	69.40
25-29.9	196	28.16
≥ 30	17	2.44
Dyslipidemia		
Yes	180	25.86
No	516	74.14

### Laboratory results of adult hypertensive patients

Patient’s laboratory result was done and results represented as follow (Table 4).

**Table 4:** Laboratory results of adult hypertensive patients attending at Comprehensive Specialized Hospital in Northwest Ethiopia 2024 (*n* = 696)

Variables	Frequency	Percentage
**Total cholesterol**		
≤ 200 mg/dl	527	75.83
> 200 mg/dl	168	24.17
**Triglyceride**		
≤ 150 mg/dl	494	70.98
> 150 mg/dl	202	29.02
**Low density lipoprotein**		
≤ 160 mg/dl	567	81.47
> 160 mg/dl	129	18.53
**Serum creatine (in mg/dl)**		
≤ 1.1	630	90.5
> 1.1	66	9.5
**Urea(BUN) in mg/dl**		
≤ 50	634	91.1
> 50	62	8.9
**Uric acid(in mg/dl)**		
≤ 7.1	643	92.4
> 7.1	53	7.6

### Ocular related data among study participants

When participants were assessed; 480(68.97%) had no history of eye examination in the last year and only 254(35.92%) had have awareness about HTN affects their eyes. Moreover, 64% of the study participants had not got physician’s advice to do eye checkup. Other ocular comorbidities were assessed with 45(6.47%) mild to severe glaucoma, 93(13.36%) dry or wet age related macular degeneration, 228(32.76%) immature cataract and 91(13.07%) diabetic retinopathy. Furthermore most of participants were visually impaired. Among visual impairments; 15.7% were severely impaired and 10.6% were blind (Table 5).

**Table 5:** Ocular related data among adult hypertensive patients attending at Comprehensive Specialized Hospitals in Northwest Ethiopia, 2024(*n* = 696)

Variables	Frequency	Percent
**Stage of HR(** ***N*** ** = 400)**		
Grade I	42	6.03
Grade II	111	15.95
Grade III	223	32.04
Grade IV	24	3.45
**Awareness about HTN that affects eye/s**		
Yes	254	36.49
No	442	63.51
**Having controlling BP to preserve vision**		
Yes	236	33.91
No	460	66.09
**History of cataract surgery**		
Yes	110	15.80
No	586	84.2
**Visual acuity status**		
6/6 - ≥6/12	106	15.2
< 6/12 -≥ 6/18	188	27.0
< 6/18 -≥60	219	31.5
< 6/60 -≥3/60	109	15.7
< 3/60	74	10.6

### Prevalence of hypertensive retinopathy

**Fig. 2:**
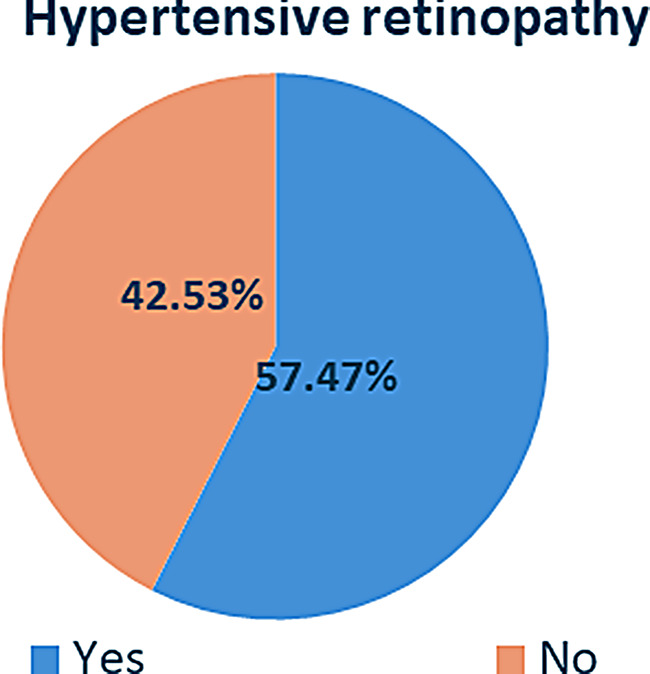
Prevalence of hypertensive retinopathy among hypertensive patient attending comprehensive specialized hospitals in Northwest Ethiopia, 2024(*n* = 696)

In this study, prevalence of HR among hypertensive patients was 57.47% (95% CI: 53.75, 61.10) (Fig. 2).

### Stages of hypertensive retinopathy

**Fig. 3:**
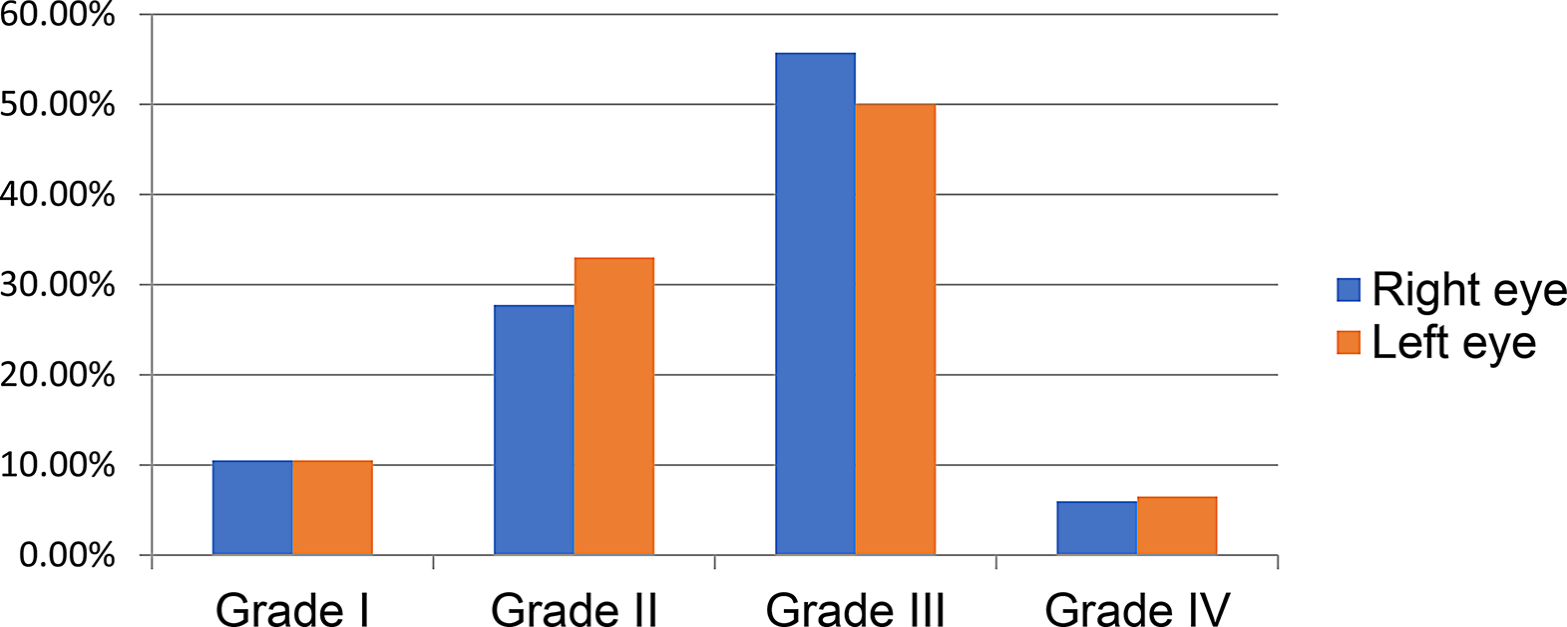
Shows grades of HR among adult hypertensive patients at Comprehensive Specialized Hospitals in Northwest Ethiopia (*n* = 400)

From HR, grade-III was high score for right eye (55.75%) and left eye (50%) (Fig. 3). This unusual condition might be due to low adherence to hypertensive medication, higher aggravating comorbidities, lower control of BP, low eye checkup practice and lack of physician advice for eye checkup were suggested.

### Factors associated with HR

By using a bivariable binary logistic regression analysis, older age, monthly income, marital status, duration of HTN, adherence of hypertensive medication, medication duration, physician advice to eye checkup, ocular examination history, current levels of HTN, DM, stroke, heart disease, chronic kidney disease, hearing HTN affecting eyes, having blood pressure control to preserve vision and BMI were independently associated with HR. However, in multivariable binary logistic regression analysis, older age, longer duration of HTN, history of heart disease, history of diabetes mellitus, history of dyslipidemia, poor adherence to hypertensive medications and uncontrolled HTN remained significantly associated with HR.

The odds of developing HR for those participants aged above 74 years were 4.24 times higher than those participants age ranges from 19-51years(AOR = 4.24, 95%CI:1.54,11.64).

Participants who had history of heart disease were 5.38 times (AOR = 5.38; 95%CI: 1.86, 15.58) more likely to develop HR than participants who had not history of heart disease. Participants with DM were 3.00 times more likely to have HR as compared with those participants without DM (AOR = 3.00; 95% CI: 1.49, 5.99).

The odds of developing HR for those participants with duration of HTN since diagnosis > 5 years were 12.66 times higher than participants with duration of HTN ≤ 3years (AOR = 12.66; 95% CI: 3.88, 41.29). Participants who had poor control of BP were 40 times more likely to have HR than participants who had good control of BP (AOR = 40.03; 95%CI: 17.19–93.18).

The odds of developing HR for those participants with dyslipidemia were 3.44 times (AOR = 3.44, 95%CI: 1.59–7.45) higher than participants without dyslipidemia.

Participants who had poor adherence of hypertensive medication were 1.84 times more likely to have HR as compare to those participants who had good adherence of hypertensive medication (AOR = 1.84, 95% CI: 1.12,3.03) (Table 6).

**Table 6:** Factors associated with hypertensive retinopathy among adult hypertensive patients attending at Comprehensive Specialized Hospitals in Northwest Ethiopia, 2024(*n* = 696)

Variables	Hypertensive retinopathy						
	Yes	No	Crude Odds Ratio(95%CI)	Adjusted Odds Ratio(95%CI)		*P*-value	
**Age(in years)**							
19–51	62	123	1.00	1.00			
52–63	77	105	1.45(0.95–2.22)	0.84(0.42–1.67)		0.62	
64–74	104	53	3.89(2.48–6.11)	1.31(0.61–2.83)		0.49	
> 74	157	15	20.76(11.27–38.27)	**4.24(1.54–11.64)**		**0.005**	
**Marital status**							
Unmarried	11	18	1.00	1.00			
Married	284	250	1.86(0.86–4.01	2.79(0.76–10.31)		0.12	
Divorced	19	13	2.39(0.85–6.70)	4.57(0.81–25.75)		0.09	
Widowed	86	15	9.38(3.70-23.76)	3.51(0.71–17.31)		0.12	
**Family average monthly income(Ethiopian birr)**							
> 12,000	74	71	1.00	1.00			
500–4148	122	52	2.25(1.42–3.57)	1.76(0.74–4.17)		0.21	
4149–8000	103	82	1.21(0.78–1.84)	0.89(0.42–1.91)		0.78	
8000–12,000	101	91	1.06(0.69–1.64)	0.86(0.42–1.78)		0.69	
**History of eye examination after diagnosis of HTN**							
Yes	164	52	1.00	1.00			
No	236	244	3.26(2.28–4.67)	1.23(0.50–3.06)		0.65	
**Duration of HTN (in years)**							
≤ 3years	59	175	1.00	1.00			
3-5years	74	95	2.31(1.51–3.53)	3.44(1.73–6.83)		**0.001**	
> 5 years	274	26	30.46(18.49–50.17)	12.66(3.8841.29)		**0.001**	
**History of heart disease**							
No	268	289	1.00	1.00			
Yes	132	7	20.33(9.35–44.28)	5.38(1.86–15.58)		**0.002**	
**History of stroke**							
No	295	289	1.00	1.00			
Yes	105	7	14.69(6.72–32.12)	2.64(0.91–7.68)		0.074	
**History of dyslipidemia**							
No	238	278	1.00	1.00			
Yes	162	18	10.51(4.33–24.65)	3.44(1.59–7.45)		**0.002**	
**History of DM**							
No	189	274	1.00	1.00			
Yes	211	22	13.90(8.63–22.39)	3.00(1.49–5.99)		**0.002**	
**Duration of using of hypertensive medication (in year)**							
≤ 5 years	143	270	1.00	1.00			
> 5 years	257	26	18.66(11.88–29.31)	1.26(0.44–3.59)		0.66	
**Current levels of HTN**							
Controlled	189	285	1.00	1.00			
Uncontrolled	211	11	28.92(15.35–54.50)	40.03(17.19–63.18)		**0.001**	
**Adherence to Hypertensive medication**							
Good	142	180	1.00	1.00			
Poor	258	116	2.81(2.07–3.85)	1.84(1.12–3.03)		0.016	
**Physicians’ advice for eye checkup**							
Yes	221	225	1.00	1.00			
No	179	71	2.57(1.84–3.58)	1.29(0.60–2.81)		0.51	
**Awareness about HTN that affects eyes**							
Yes	226	216	1.00	1.00			
No	174	80	2.08(1.50–2.87)	1.85(0.62–5.48)		0.27	
**Having control BP to preserve vision**							
Yes	235	216	1.00	1.00			
No	165	80	2.23(1.60–3.16)	1.1.25(0.45–3.48)		0.66	

## Discussion

The prevalence of HR in this study was 57.47% (95% CI: 53.75, 61.10) which in lines with un observational study done in Pakistan 56% [24] and hospital based cross-sectional study done in Nepal 56.5% [29]. This might be due to similarities of socio-demographic factors like age of participants.

However, the result of this study was lower than cross-sectional studies conducted in Democratic Republic of Congo 83.6% [42], Tanzania 70.1% [22], Malawi 75% [43], Nigeria 71% [44], China 77.1% [26], Nepal 83.7% [28].

This discrepancy could be due to differences in the socio-demographic, clinical characteristics of the study population, the study area and the newly diagnosed hypertensive patients. For example, the studies in Democratic Republic of Congo and Nigeria were conducted in hypertensive patients with cardiovascular disease; on the other hand, this study was conducted on hypertensive patients with or without having comorbidities on hypertensive patient. A study done in Tanzania was on hypertensive patients with chronic kidney disease, and study conducted in China on hypertensive patients with albuminuria but this study was conducted on patients with/without comorbidities. Those conditions are risk factors for high magnitude of HR; and might be the cause for the discrepancy.

In addition, the study conducted in Nepal was specifically referred to an age ≥ 60 years, which increases the magnitude of HR, but in our study the population was age ≥ 18 years old. Moreover, a study done in Pakistan was on inpatient wards, which increases HR prevalence but our study was on outpatient hypertensive patients by excluding inpatient hypertensive participants. Finally, a study conducted in China was on newly diagnosed hypertensive patients, which exaggerates the magnitude of HR.

On the contrary, the finding of this study was higher than cross-sectional studies done in Iran 39.9% [11], Nepal 12.6% [30], Denmark 8.3% [38], Bangladesh 29.9% [25], Cameroon 16% [40] and Kenya 23.3% [41].

This discrepancy could be due to differences in study population characteristics, such as duration of hypertension, blood pressure control status, and differences in study settings. For example, a study done in Nepal was a population-based study, while this study was conducted at chronic follow up clinical.

Studies in Kenya, Cameroon, India, Iran and Bangladesh were conducted in one hospital setting, but this was done at multicenter leading to high extent of HR prevalence.

In addition, the studies conducted in the US, Denmark and Texas were carried out on hypertensive patients without diabetes and coronary heart disease, but this study included these patients which are important risk factors for the development HR [61, 62],whereas this study tried to include all hypertensive patients with or without concomitant systemic diseases like DM or coronary heart disease. This could be the reason for the high prevalence of HR in this study.

Moreover, participants from US, Denmark, Texas, Iran and others are from developed countries which had better setup for earlier diagnosis and giving awareness about HTN, but in this study earlier diagnosis opportunity is low and hospitality setup is retarded relative to others which impacts high magnitude of HR.

In this study, participants aged over 74 years had a 4.24-fold higher risk of developing HR than participants aged 19–51 years. This finding was consistent with studies done in Turkey [63], Iran [11], China [19], Bangladesh [25] and India [34]. This is due to the stiffening of arteries caused by the gradual replacement of elastin by collagen and other age-related changes [64]. When the large arteries are affected by stiffening, the aortic reservoir cannot expand and store as much blood [65].

This study showed that participants with duration of HTN of 3–5 years and over 5 years had a 3.44- and 12.66-fold higher risk of HR, respectively, than participants with duration of HTN ≤ 3 years. This finding is supported by the studies done in Bangladesh [25], India [66, 67], and Nepal [68]. This is because persistent high BP leads to exudative vascular changes, which is a consequence of endothelial damage and necrosis [69].

In the present study; participants with uncontrolled HTN were forty times (40.03) more likely to develop HR than participants who had their BP under control. This study was consistent with the studies done in Turkey [70], Island [47], Sidney [69] and China [19].

This is because increased BP is transmitted directly to the vessels, which initially constrict the retinal vessels. However, further increase in BP overcomes this compensatory tone, leading to damage to the muscle layer and the endothelium [71]. For this reason, severe or prolonged HTN disrupts the blood‒retinal barrier, leading to necrosis of retinal endothelial cells, exudation of blood and lipids, and ischemia of the retinal nerve fiber layer [72].

In addition, arterioles of the retina will show an increased light reflex when BP is uncontrolled. Over time, the interface between the arteriole and the vein changes, with the arteriole pressing on the vein and causing compression at the interface. If the stress on the vascular system is not stopped, loss of auto-regulation can occur, leading to unexpected loss of arteriole and endothelial cells. There is leakage of plasma and blood from the vessels, including exudates [70]. Finally, after an increase in BP, the microcirculation of the retina is damaged to varying degrees.

In current study, participants with HTN with dyslipidemia had a 3.44 times higher risk of developing HR than participants without a history of dyslipidemia. The result was supported by the studies done in Nepal [29, 73], Moldova [74] and India [75].

The possible reason for this is that high BP causes the blood to be forced through the vessels at a higher pressure, while dyslipidemia increases the viscosity of the blood. Cholesterol emboli may be released from plaques in the internal carotid artery and travel into the arterioles of the retina [70].

The other reason is that a high concentration of cholesterol in the blood in the form of low-density lipoproteins is the primary causal factor for atherosclerosis, which affects the larger caliber arteries and the central retinal artery before it branches, while arteriolosclerosis develops in the retinal arterioles [75].

This study found that participants with a history of heart disease were 5.38 times more likely to develop HR than participants without a history of heart disease. This study result was agreed with studies done in Australia [76], Turkey [77], Japan [39, 78], German [79],and Netherland [80].This could be due to the fact that elevated BP increases luminal pressure and activates the auto-regulatory mechanism responsible for the narrowing of the arteries and the occurrence of vasospasm. This is directly related to cardiovascular disease and increased arteriolar wall reflex within the joint adventitial sheath due to increased BP [78].

On the contrary, other previous studies have shown that the relationship between heart rate and the incidence of heart disease is independent of HTN. Although the underlying mechanism is not yet fully understood, unusual HTN that are not revealed by typical measurements, such as paroxysmal and nocturnal HTN and microarterial HTN, play an important role [39]. HR is independently associated with a higher risk of cardiovascular disease regardless of BP and other cardiovascular risk factors [81].

Study participants who had DM were three times more likely develop HR than those participants who were not DM. This finding was consistent with the studies done in Nepal [68] and US [82]. This might be due to the endothelium of the retinal capillaries is injured in HR, and diabetes promotes endothelial disturbance [83].

Moreover, participants who had poor medication adherence were 1.84 times more likely to develop HR than those participants who had good medication adherence. Patients poor adherence in patients of HTN who perceived adverse effects [84]. This is due to significant relationship between poor adherence and uncontrolled HTN [85]. So, this uncontrolled HTN leads to formation of HR.

### Limitation of the study

The limitations of the study include: Being a cross-sectional study, this study may show the temporal relationship between predictors and hypertensive retinopathy rather than the actual cause–effect relationship. Also, this study was conducted in three specialized hospitals in Northwest Ethiopia, which may affect the generalizability of the study results to the entirety of hypertensive patients in Ethiopia. In addition, electrolyte testing was not performed on the participants of this study for data consistency, as this was not done in all hospitals.

## Conclusion

The prevalence of hypertensive retinopathy is high among systemic hypertensive patients seen in Northwest Ethiopia comprehensive specialized hospitals and independently associated with older age, longer duration of hypertension, heart disease, diabetes, dyslipidemia, poor adherence of hypertension medications and uncontrolled hypertension. Early diagnosis and treatment of hypertension was recommended to prevent target organ complications.

### Recommendations

#### For ministry of health

It is better to educate the population about the effects of hypertensive retinopathy and to take measures for prevention and treatment.

#### For University of Gondar, Felege and Tibebe Ghion Comprehensive Specialized hospitals

It would be better to make hypertensive retinopathy an integral part of the eye examination and create awareness of the ocular complications of hypertension and the effects of medication use to prevent these complications in patients.

#### For physicians

It is better if hypertensive patients are treated holistically by a doctor and an ophthalmologist together to prevent visual impairment due to hypertensive retinopathy.

#### For participants

It is better to control high blood pressure and perform regular follow-up examinations to avoid hypertensive retinopathy.

#### For researchers

Finally, the researchers had to conduct further longitudinal studies to measure the causal relationship of hypertensive retinopathy.

## Data Availability

No datasets were generated or analysed during the current study.

## Abbreviations

AOR: Adjusted Odd Ratio
AVN: Arterio-Veinous Nicking
BMI: Body Mass Index
BP: Blood Pressure
CI: Confidence Interval
DM: Diabetes Mellitus
D: Diopter
HR: Hypertensive Retinopathy
HTN: Hypertension
IQR: Inter Quartile Range
KG: Kilogram
MmHg: Millimeter of Mercury
TOD: Target Organ Damage
USA: United States of America
VIF: Variance Inflation Factor
